# Highlighting the hidden: monitoring the avidity-driven association of a fluorescent GABARAP tandem with microtubules in living cells

**DOI:** 10.1080/27694127.2024.2348899

**Published:** 2024-05-16

**Authors:** Alina Üffing, Lisa Gold, Thomas Gensch, Oliver H. Weiergräber, Silke Hoffmann, Dieter Willbold

**Affiliations:** a Heinrich-Heine-Universität Düsseldorf, Mathematisch-Naturwissenschaftliche Fakultät, Institut für Physikalische Biologie, Düsseldorf, Germany; b Forschungszentrum Jülich, Institut für Biologische Informationsprozesse: Strukturbiochemie (IBI-7), Jülich, Germany; c Forschungszentrum Jülich, Institut für Biologische Informationsprozesse: Molekulare und Zelluläre Physiologie (IBI-1), Jülich, Germany

**Keywords:** ATG8, bivalence, live-cell imaging, tubulin, mTagBFP2, tagging

## Abstract

GABARAP, like other ATG8 proteins, is a ubiquitin-like modifier and its C-terminal lipid conjugation enables association with cellular membranes. To prevent interference with the lipidation process, N-terminal fluorescent protein (FP) tagging strategies have become the standard for studying ATG8 localization and function in living cells, significantly contributing to our understanding of this protein family’s multifaceted roles. We employed live cell imaging with particular emphasis on a GABARAP split-tandem construct, GABARAP(G116A)-mTagBFP2-GABARAP (G-*B*-G), which retains both a free N-terminus and a lipidation-competent c-terminus, while bivalence creates a gain in affinity conferred by avidity. Notably, reminiscent of early *in vitro* studies demonstrating an interaction of GABARAP and tubulin, our results revealed a robust association of G-*B*-G with the microtubule network in living cells. We show that the presence of several basic residues in the amino-terminal helical subdomain of GABARAP and avidity emerged as essential for robust MT association, whereas lipidation ability was not decisive. Interestingly, while the position of the FP-tag had little influence on the result, the nature of the FP itself was crucial, with mTagBFP2 being required for tracking GABARAP tandems in the vicinity of MTs. Though artificial effects cannot be excluded, we assume that G-B-G, with its increased avidity, can give visibility to processes that are based on inherently weak interactions, and thus can help elucidate potential roles of GABARAP e.g. in microtubule-associated processes that are integral to autophagy-related and -unrelated cellular transport.

## Introduction

The γ-aminobutyric acid type A receptor associated protein GABARAP belongs to the autophagy related protein 8 (ATG8) family, which in humans comprises a total of seven proteins. Human ATG8s are commonly split into two subfamilies, one containing the microtubule-associated protein 1 light chain 3 (MAP1LC3, hereafter referred to as LC3) isoforms LC3A, LC3B, LC3B2, LC3C, and one containing GABARAP, GABARAPL1, GABARAPL2. Like all ATG8 proteins, GABARAP consists of two N-terminal alpha-helices followed by a ubiquitin-like fold, and can similarly to ubiquitin be covalently conjugated to a substrate by an E1-E2-E3-like enzyme cascade [1–5]. Prior to substrate conjugation, ATG8s’ C-termini require processing by an ATG4 cysteine protease resulting in the exposure of a terminal glycine residue (e.g. G116 in GABARAP or G120 in LC3B). Deconjugation is also mediated by ATG4 proteases, making the process reversible [6-8]. Unlike ubiquitin, which is conjugated to amino groups in proteins, ATG8s usually exploit phosphatidylethanolamine (PE) or -serine (PS) as substrates, localizing them to membranes when lipidated [9]. However, exceptions have recently been described for both types of modifiers [10–12].

To avoid conflict with the cellular lipidation process, N-terminal tagging strategies have been highly recommended for all ATG8s in the past [13], because C-terminal tagging would either result in loss of the tag or, in case of ATG4 processing-resistant ATG8 mutants like GABARAP(G116A), prevent membrane conjugation [13,14]. ATG8s with N-terminal FP tags are for instance widely used to monitor autophagy [15–17], and in conjunction with other techniques, have helped to elucidate the versatile roles of ATG8 proteins and shaped the current understanding of this multi-facetted protein family, which has been associated with a, still growing, plethora of autophagy-related and -unrelated functions [18–21].

Interestingly, most of the underlying mechanisms have in common that they involve direct interactions of the ATG8s with other proteins. In the majority these interactions rely on a conserved motif in the interaction partner (LC3-interacting region, LIR; ATG8/GABARAP-interacting motif, AIM/GIM) and its corresponding docking site (often termed LDS for LIR-docking site) spanning two hydrophobic pockets on the ATG8 protein surface [22,23].

While it appears that both the lipidation machinery and many LIR-LDS-mediated ATG8-associated processes can tolerate the presence of bulky, and even multiple, N-terminal tags [24], a growing body of evidence demonstrates that common ATG8 constructs, with bulky FP tags at least twice their size, can be functionally compromised and/or mislocalized. One example are studies on mitophagy, where N-terminal FP-tags were detrimental to GABARAP’s localization to mitochondria [25,26], while the smaller haemagglutinin-tag (HA-tag) did not inhibit mitochondrial targeting [26]. The unique N-terminal region of ATG8 proteins distinguishes them from other ubiquitin-like proteins and its primary structure also varies remarkably among the different GABARAPs/LC3s. Notably, structural biology studies indicate that, contrary to the conserved and rather rigid ubiquitin-like cores, the N-termini of several human ATG8s, like the yeast Atg8 ancestor, exhibit high flexibility [1,27-29]. The N-terminal region of GABARAP, for instance, is known to adopt multiple conformations [1,30] and has been described to be involved in its self-association [31,32], in the regulation of its proteasomal degradation through MIB1-mediated ubiquitination of K13 and K23 [33], and in its membrane association [34]. Historically, one of the first described functional features of the GABARAP N-terminal region was its tubulin and microtubule (MT) binding activity *in vitro* [35,36], albeit affinities between GABARAP and tubulin-derived peptides spanning the tubulin C-termini have been considered low [30]. As appropriate data in the cellular context are sparse [36], further investigation of GABARAP’s MT association in living cells is still pending. Since systematic studies on ATG8-tagging strategies in general have received little attention in the past, we sought to fill this gap by employing a live cell imaging approach using cells expressing different FP arrangements of GABARAP. In contrast to the popular FP-GABARAP orientation, the presented work focuses on a so-called GABARAP split-tandem construct, GABARAP(G116A)-mTagBFP2-GABARAP (G-*B*-G). In G-*B*-G, an ATG4-resistant GABARAP with G116A substitution is joined to a central FP (mTagBFP2) followed by another GABARAP. In addition, its bivalence creates a gain in affinity conferred by avidity, potentially visualizing localizations of GABARAP which otherwise, presumably due to low-affinity interactions, might be hidden.

## Material & methods

### DNA constructs

Plasmids used throughout this study are listed in Table 1. All newly introduced vectors were generated by restriction-ligation cloning into pcDNA5/FRT/TO. Synthetic DNA fragments encoding GABARAP(G116A)-mTagBFP2-GABARAP (G-*B*-G), GABARAP(5X/G116A)-mCherry-GABARAP (G^5X^-*mCh*-G), GABARAP(G116A)-mTagBFP2-GABARAP(5X) (G-*B*-G^5X^) and GABARAP(G116A)-mTagBFP2 (G-*B*) were obtained from Geneart and BioCat. All used constructs were sequence verified (Microsynth Seqlab). The respective plasmid DNAs have been deposited at the Addgene plasmid reposatory, where more detailed information for each construct can be accessed.

**Table 1.** List of constructs used in this work, together with short names, vector backbone, protein encoded, details (e.g. position-specific amino acid substitutions), and reference or source. Abbreviations: G: GABARAP; B: mTagBFP2; mCh: mCherry; Y: eYFP; *: G116A or G120A.

Short names	Vector	Encoding	Details	Reference/Source
G-*B*-G	pcDNA5/FRT/TO	GABARAP(G116A)-mTagBFP2-GABARAP		Addgene #212106
G^5X^-*B*-G	pcDNA5/FRT/TO	GABARAP(5X/G116A)-mTagBFP2-GABARAP	5X: K2, K13, R15, K20, K23 to A	Addgene #212107
G-*B*-G^5X^	pcDNA5/FRT/TO	GABARAP(G116A)-mTagBFP2-GABARAP(5X)	5X: K2, K13, R15, K20, K23 to A	Addgene #218425
G^5X^ -*B*-G^5X^	pcDNA5/FRT/TO	GABARAP(5X/G116A)-mTagBFP2-GABARAP(5X)	5X: K2, K13, R15, K20, K23 to A	Addgene #218426
G-*B*-G*	pcDNA5/FRT/TO	GABARAP(G116A)-mTagBFP2-GABARAP(G116A)	lipidation-deficient	Addgene #212108
*B-*G-G	pcDNA5/FRT/TO	mTagBFP2-GABARAP(G116A)-GABARAP		Addgene #212109
*B*-G	pcDNA5/FRT/TO	mTagBFP2-GABARAP		Addgene #212110
*Y*-G	pcDNA5/FRT/TO	eYFP-GABARAP		Addgene #212111
G-*B*	pcDNA5/FRT/TO	GABARAP(G116A)-mTagBFP2		Addgene #212112
G	pcDNA5/FRT/TO	GABARAP	tag-less	Addgene #218427
G-*mCh*-G	pcDNA5/FRT/TO	GABARAP(G116A)-mCherry-GABARAP		Addgene #212113
mEos-tubulin	mEos3.2-C1	mEos3.2-Tubulin-C-18		Addgene #57484

### Cell culture and transfection

Human hepatoma Huh-7.5 *GABARAP* KO cells, HEK293 Flp in TRex *GABARAP* KO cells [37] and HeLa cells were cultured in Dulbecco’s Modified Eagle Medium (DMEM) high glucose (Sigma Aldrich, D5796), supplemented with 10% heat inactivated fetal bovine serum (Sigma Aldrich, F9665) and 1% penicillin/streptomycin (Sigma Aldrich, P4333) at 37°C and 5% CO_2_. Cells were passaged at 80% confluency and routinely checked for mycoplasma contamination.

For transfection, cells were grown in 6- or 12- well plates to at least 70% confluency and transfected with 3 or 1.5 µg plasmid DNA per well, using 9 or 4.5 µl Lipofectamine 2000 (Thermo Fisher Scientific, 11668019) respectively. Cells were seeded into fibronectin (Sigma Aldrich, F1141) coated 35 mm glass-bottom IBIDI dishes 1.5-3 h post transfection and either imaged after 24-42 h or fixed for immunocytochemistry.

### Immunocytochemistry

Transfected Huh-7.5 *GABARAP* SKO cells were fixed for 10 min with precooled methanol and subsequently for 1 minute with precooled acetone, both at -20°C. After two washes with PBS (137 mM NaCl, 2.7 mM KCl, 1.8 mM KH_2_PO_4_, 10 mM Na_2_HPO_4_, pH 7.4), non-specific binding sites were blocked with 5% (w/v) BSA (AppliChem, A1391) in PBS for 1-2 h. Cells were incubated with primary antibody (anti-GABARAP rabbit polyclonal [Proteintech, 18723-I-AP) diluted 1:100 in PBS containing 1% (w/v) BSA and 0.3% (v/v) Triton X-100 (AppliChem, A4975) for 2 h or overnight and afterwards washed twice with PBS. Cells were stained with secondary antibody (anti-rabbit-Alexa647 [abcam, ab150083]) diluted 1:200 in the abovementioned solution for 1 h. Following two washes with PBS, cells were imaged and stored in PBS containing 0.05% (w/v) sodium azide.

### Confocal Laser Scanning Microscopy

Cells were imaged using a LSM 710 confocal laser scanning system (Zeiss), operated with ZEN black 2009 software and a Plan-Apochromat 63x/1.40 Oil DIC M27 objective. For live-cell imaging at 37°C, a temperature-controlled microscopy stage was used. For staining of actin and microtubules, medium was supplemented with 500 nM SiR-tubulin (Spirochrome, SC002) or 1 µM SiR-actin (Spirochrome, SC001) and cells were incubated for 4 to 8 h prior to imaging. In the case of Huh-7.5 *GABARAP* KO and HEK293 Flp in TRex *GABARAP* KO, cells were additionally treated with 10 µM verapamil. The laser excitation wavelength and emission filters for the cells expressing fusion protein constructs were 405 nm and 410-509/530 nm for mTagBFP2, 514 nm and 519-621 nm for eYFP, 543 nm and 578-696 nm for mCherry and 633 nm and 638-759 nm for cells stained with SiR-probes.

### Colocalization colormap

To obtain a spatial representation of the colocalization of G-*B*-G with SiR-tubulin or SiR-actin we used the “colocalization colormap” Plugin for ImageJ [38], which is based on the Jaskolski algorithm [39]. This method creates a pseudo-color map of correlations between pairs of corresponding pixels in two input images. As input for the analysis we used smoothed images (3x3 mean filter) of G-*B*-G-expressing Huh-7.5 *GABARAP* KO cells counter-stained either with SiR-tubulin or SiR-actin. Per analysed cell, five regions of interest (ROIs) of 40×40 pixels were split into their corresponding mTagBFP2- and SiR-channels. For each ROI the normalized mean deviation product (nMDP) was calculated for all corresponding pixels in the two channels, mathematically representing correlation between intensities of each pixel pair. The distribution of the calculated nMDP values was plotted, resulting in a colocalization colormap for each ROI. Such maps display the spatial correlation between the two fluorescent signals (mTagBFP2 and SiR), and because a jet colormap is implemented in the plugin by default, hot colours indicate colocalization and cold colours indicate separation. As a quantitative measure, the plugin also calculates the index of correlation (Icorr), which represents the fraction of positively colocalized pixels in each analyzed ROI. Finally, in order to compare the results, the distribution of Icorr values obtained for either G-*B*-G and SiR-tubulin or G-*B*-G and SiR-actin were plotted separately, and were subjected to statistical evaluation.

### Quantitative analysis of N:C, Nu:N and F:C ratios

To quantify the nucleocytoplasmic (N:C) ratio, five randomly ROIs (⌀ 10 px) were manually drawn in the nucleus as well as in the puncta-devoid cytoplasmic region of each cell using ImageJ. Mean intensities of nuclear and cytoplasmic ROIs from mTagBFP2 and eYFP channels were measured and divided. Likewise, mean intensities from 2-3 nucleoli ROIs were measured and divided by the mean nuclear intensity for both channels to determine the nucleolar to nucleoplasm (Nu:N) ratio of each cell. Filament to cytoplasm (F:C) ratios were determined by selecting 2-3 regions surrounding filaments using the polygon selection tool in ImageJ. For each filament ROI, a corresponding ROI of the same shape and size was placed in a nearby cytoplasmic region without filaments. Mean intensities were measured and F:C ratios calculated per ROI pair and cell. In case of co-transfected cells, this was done for both the mTagBFP2 and the eYFP channel. In case of single construct transfection and co-staining with SiR-tubulin, ROI pairs were selected according to most intense signals in the SiR channel and mean intensities were measured from the mTagBFP2 channel. N:C, Nu:N and F:C ratios were plotted and subjected to statistical evaluation.

### Microtubule organization analysis

For analysis of the microtubule cytoskeleton, images showing a G-*B*-G expressing and an un-transfected control cell (co-)stained with SiR-tubulin were selected. The areas of both individual cells were manually traced according to the transmission image and saved as ROIs. SiR channels were skeletonized using the ImageJ LPX filter2D plugin (filter = lineFilters, linemode = lineExtract, giwsiter = 5, mdnmsLen = 15, pickup = otsu, shaveLen = 5, delLen = 5) [40]. Afterwards, the skeleton image type was set to 8 bit, duplicated, ROIs selected and background set to black (0,0,0) to obtain a skeletonized microtubule image per cell (ROI). Subsequently, the skeleton of each cell was analysed using ImageJ (Analyze > Skeleton) to tag endpoint, junction and slab pixels (the pixels between junctions and endpoints). Length of branches above 3 pixels was plotted for each cell in µm.

Additionally, cytoskeleton bundling parameters (Skewness and coefficient of variation [CV]) were obtained from the skeleton images of each cell according to an ImageJ macro published by [41], modified for analysis of single plane images.

### Prediction of complex structures

Potential modes of interaction of human GABARAP or its mTagBFP2 tandem constructs with microtubules were investigated *in silico* with AlphaFold2 [42], using the ColabFold implementation [43]. Complexes of GABARAP and a TBA1A-TBB5 (Uniprot accession Q71U36, P07437) dimer were predicted using a local installation (github.com/YoshitakaMo/localcolabfold) running on a Linux workstation equipped with an nVidia GPU, while data for the larger complexes of G-*B*-G or *B*-G-G with a tetrameric tubulin chain (TBA1A-TBB5-TBA1A-TBB5) were uploaded to the COSMIC^2^ platform [44] for processing by ColabFold. For each subject, five models were generated with inclusion of template information, using multiple sequence alignment mode *mmseqs2_uniref_env*, model type *alphafold2_multimer_v3*, a maximum of 20 recycle steps, and recycling controlled by *recycle_early_stop_tolerance:0.5* and *stop_at_score:90* for the local and remote installations, respectively. All models were subjected to relaxation using Amber as implemented in the ColabFold pipeline. The highest ranked model without steric clashes, according to predicted lDDT statistics, is used for further evaluation; the complete list of models along with predicted lDDT and PAE plots is shown as supplemental information (Fig. S5). Figures were created using the PyMOL Molecular Graphics System, Version 2.7 Schrödinger, LLC.

### Statistical analysis

Mean N:C and Nu:N values per cell as well as resulting statistics from paired t-tests were plotted using GraphPad Prism version 9. Mean F:C values per cell were analyzed by paired t-test in case of co-transfected cells and one-way Anova with Tukey’s multiple comparison test for single transfections with different constructs using GraphPad Prism version 9, and mean values as well as individual values were plotted using SuperPlot (https://huygens.science.uva.nl/SuperPlotsOfData/). Mean Icorr values per cell were analyzed by unpaired t-test, and individual values as well as means were plotted using GraphPad Prism 9 and SuperPlot, respectively. Branch length was analyzed for each cell pair using Mann-Whitney test, and individual values were plotted with GraphPad Prism 9.

## Results

### G-*B*-G exhibits subcellular localization distinct from *Y*-G

**Figure 1:**
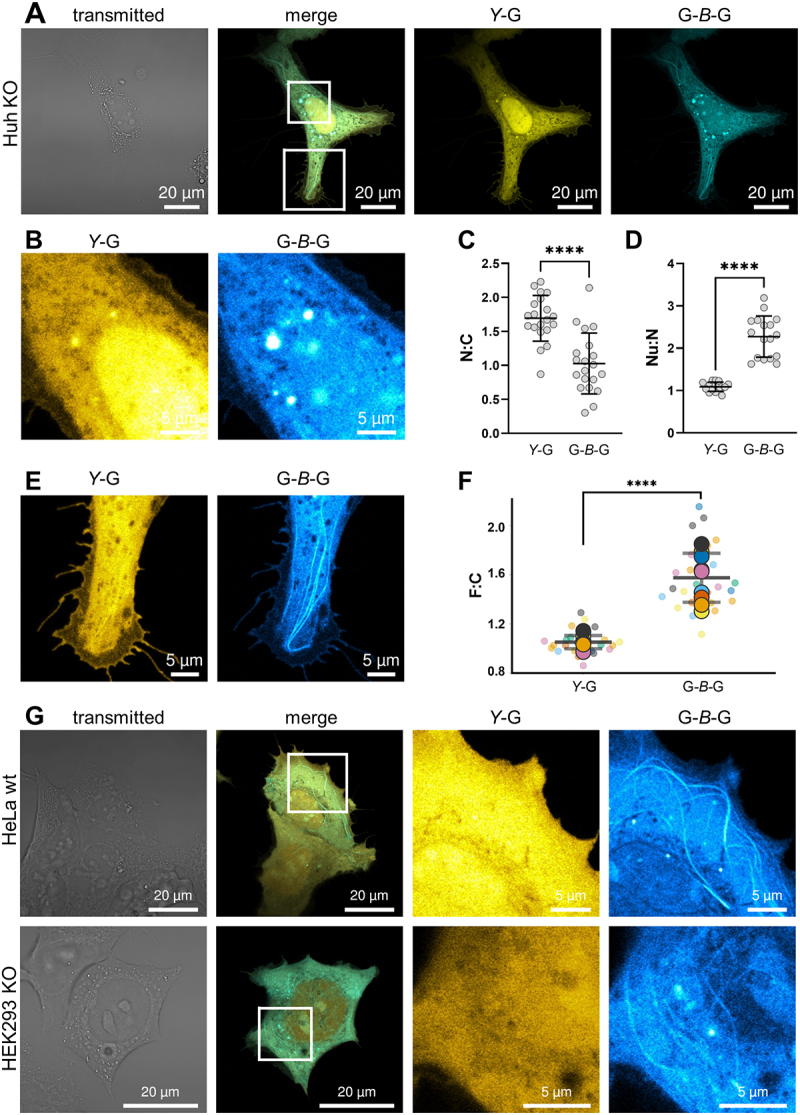
Distinct subcellular distribution of *Y*-G and G-*B*-G. (**A**) Exemplary image of a Huh-7.5 GABARAP KO cell expressing G-*B*-G and *Y*-G. Distinct signals are visible at different subcellular locations including (**B**) nucleus and nucleoli and (**E**) filaments. Quantification of (**C**) nucleocytoplasmic ratio (N:C), Mean: 1.03 ± 0.45 for G-*B*-G and 1.69 ± 0.34 for *Y*-G; (**D**) Nucleoli to nucleoplasm (Nu:N) ratio, Mean: 2.27 ± 0.49 for G-*B*-G and 1.09 ± 0.11 for *Y*-G and (**F**) filament to cytoplasm ratios (F:C), Mean: 1.57 ± 0.2 for G-*B*-G and 1.05 ± 0.05 for *Y*-G measured by mean fluorescence intensity. **** P < 0.0001, paired t-test. Values are represented as means ± SD (n= 20 (N:C), 17 (S:N), 9 (F:C)) from two independent experiments). See Fig. S1A for a detailed description of the quantification procedure applied. All cells and ROIs that went into the quantification can be reviewed on BioImage Archive. (**G**) In HEK293 GABARAP SKO and HeLa wildtype cells, distinct features can also be observed for overexpressed G-*B*-G compared to *Y*-G.

Due to Atg8-like proteins being C-terminally conjugated to lipids, their N-terminal fusions with fluorescent proteins (FP-ATG8s), such as the yellow fluorescent protein-tagged GABARAP (*Y*-G) used in this study, have a long tradition in the study of their function in living cells. Macroautophagy/autophagy induction typically triggers FP-ATG8s to localize to punctate structures, interpreted as their lipidated forms associated with autophagic membranes. Under basal conditions, being the focus here, FP-ATG8s are known for their diffuse cytoplasmic and nuclear distribution - commonly interpreted as their free, unlipidated forms. To restrict GABARAP functionalities to the transfected constructs, excluding any contribution from an endogenous GABARAP background, Huh-7.5 *GABARAP* knockout cells, hereafter abbreviated as Huh KO cells, were used throughout this study unless otherwise stated. In order to be able to directly compare the behavior of the novel split tandem construct G-*B*-G with that of *Y*-G under basal conditions, cells were co-transfected with the corresponding expression plasmids (Table 1). Overexpressed *Y*-G showed, as expected, a diffuse cytoplasmic distribution and high intensities in the nucleoplasm, however, co-expressed G-*B*-G emerged at a variety of intracellular structures, resulting in a more heterogenous staining pattern (Figure 1A). Even though the mean nucleocytoplasmic (N:C) ratio of *Y*-G (1.69 ± 0.34) was significantly higher than for G-*B*-G (1.03 ± 0.45), the latter showed relatively high intensities at diverse nuclear subcompartements including the nucleolus, with a higher mean nucleoli-to-nucleoplasm (Nu:N) ratio (2.27 ± 0.49) compared to *Y*-G (1.09 ± 0.11; Figure 1B–D).

Most strikingly, G-*B*-G intensity was frequently high at filamental structures while those were barely visible in the yellow channel *(Y*-G) in the respective cells (Figure 1E). This is also reflected in the corresponding F:C ratios, which were significantly higher in the case of G-*B*-G (*Y*-G: 1.05 ± 0.05, G-*B*-G:1.57 ± 0.2; Figure 1F). To assess whether the discrepancies in signal distribution between G-*B*-G and *Y*-G arose from bivalence of G-*B*-G, the different FPs used, and/or their position in the fusion proteins, Huh KO cells were transfected with additional constructs expressing N- or C-terminal fusions of mTagBFP2 with GABARAP (*B*-G or G-*B*) as well as tandem GABARAP with N-terminal mTagBFP2 (*B*-G-G). For high nucleolar signal intensities, the FP seems to be decisive as cells expressing *Y*-G did not but *B*-G did highlight nucleoli in a manner seen in G-*B*-G expressing cells (Fig. S1B-D). In contrast to G-*B*-G, *B*-G transfected cells showed no apparent filamentous pattern. In addition, in cells expressing a C-terminally tagged, lipidation-deficient GABARAP with a free and unaltered N-terminus (G-*B*) no robust filamentous pattern could be monitored either (Fig. S1E), suggesting that the mere presence of an unmodified N-terminus is not sufficient to establish filament association of a single, FP-linked GABARAP or at least fails to achieve the degree of association sufficient for a robust microscopic detection. Notably, N-terminal FP-tagging of two consecutive GABARAPs, as shown for *B*-G-G transfected cells (Fig. S1F), resulted in the appearance of filaments reminiscent in extent of those in G-*B*-G transfected cells. Thus, enhancing affinity through bivalence by combining two GABARAP moieties in a single FP-fusion appears to be critical for highlighting filaments in living cells.

Notably, this phenomenon and the other distinct features of G-*B*-G and *Y*-G described above were also evident in other lines as illustrated for HEK293 cells in a GABARAP single-KO background, and for wildtype HeLa cells (Figure 1G). However, since we observed pronounced G-*B*-G-decorated filaments particularly frequently in Huh KO cells, this line was used for all further experiments.

### G-*B*-G-decorated filamental structures correspond to microtubules

**Figure 2:**
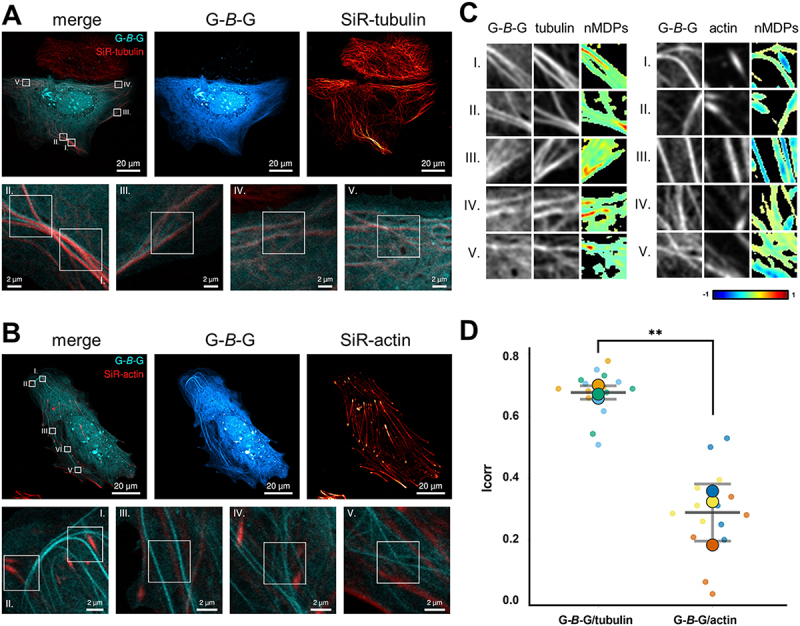
Filamental G-*B*-G structures correlate with SiR-tubulin signal. Representative live-cell images of G-*B*-G expressing Huh KO cells stained with (**A**) SiR-tubulin or (**B**) SiR-actin. Bottom panels show magnifications around five selected ROIs per cell. (**C**) Grey-scale images of the ROIs depicted in (A) and (B) highlighting the distribution pattern of G-*B*-G and tubulin (left) or actin (right). The corresponding colocalization colormaps (nMDPs, 40x40 pixel) show the spatial correlation between the two fluorescent signals (mTagBFP2 and SiR), with hot colors indicating colocalization and cold color indicating separation (n=3; refer to Fig. S3 for replicates). (**D**) Graphical representation of the corresponding Icorr values representing the fraction of positively correlated pixels for G-*B*-G and tubulin or actin. Values are plotted both for each ROI (small dots; Icorrs of the same cell have the same color) and as mean Icorr for all ROIs of a single cell (large dots). From the latter the overall Icorr mean and SD for G-*B*-G and tubulin (0.68 ± 0.02) and G-*B*-G and actin (0.29 ± 0.09) were calculated. ** P=0.0021; unpaired t-test.

To investigate the identity of the G-*B*-G enriched filamental structures, Huh KO cells expressing G-*B*-G were co-stained with SiR-tubulin and SiR-actin. Representative images showed a broad match of the fluorescence signals for G-*B*-G with SiR-tubulin (Figure 2A, Fig. S2A), whereas G-*B*-G and SiR-actin signals overlapped poorly, at best appearing with a parallel offset or crossing each other (Figure 2B, Fig. S2B). Spatial correlation of G-*B*-G with SiR-tubulin and SiR-actin signals was quantitated as outlined in the Methods section; Icorr values determined for G-*B*-G and tubulin (0.68 ± 0.02) were significantly higher than those for G-*B*-G and actin (0.29 ± 0.09), confirming the abovementioned observation (Figure 2C–D). In most cases the enrichment of G-*B*-G along MTs was uniform, but in some cells G-*B*-G positive puncta, possibly G-*B*-G decorated transport vesicles, were observed in proximity to MTs (Fig. S2C, D), raising the idea of a connection between vesicle-associated GABARAP and MTs.

### Enrichment of G-*B*-G at microtubules relies on two intact GABARAP N-termini while lipidation capability is dispensable

**Figure 3:**
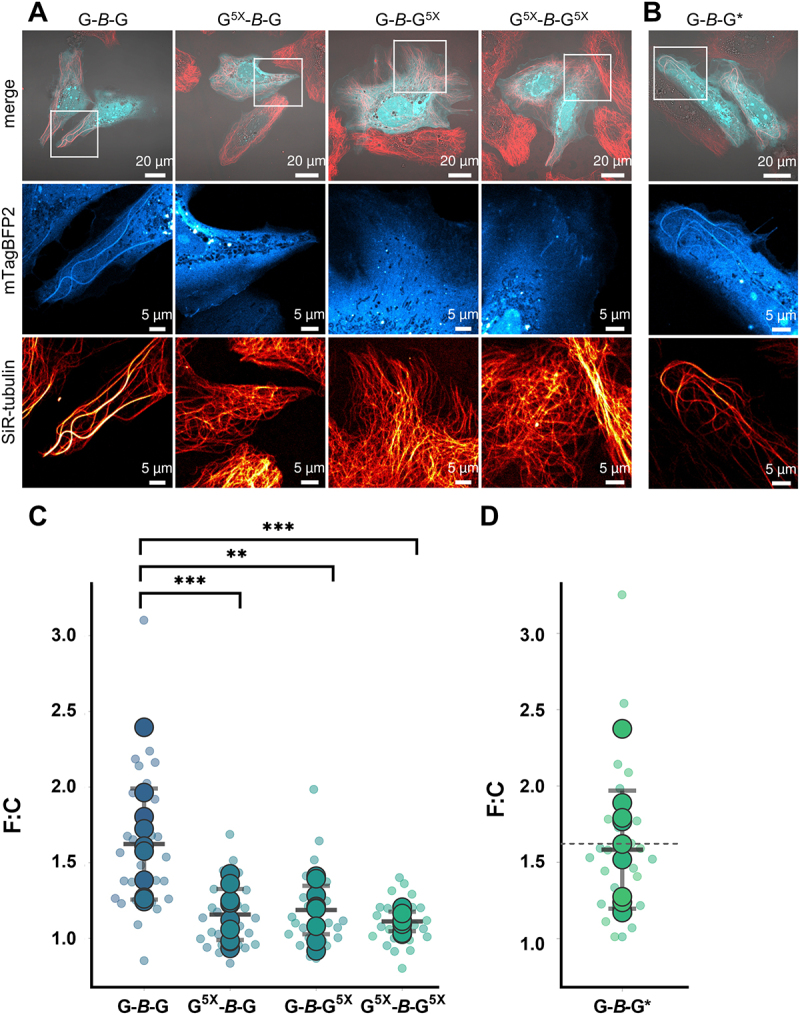
Reduced filament/microtubule association in the absence of conserved, positively charged residues in the tubulin-binding region. Exemplary images of Huh KO cells stained with SiR-tubulin expressing (**A**) G-*B*-G, G^5X^-*B*-G, G-*B*-G^5X^, G^5X^-*B*-G^5X^ and (**B**) G-*B*-G* (**C**, **D**) Quantification of filament to cytoplasm ratios (F:C) measured by mean mTagBFP2 fluorescence intensity at respective ROIs with strong SiR-tubulin signal (2-3/cell, small dots, mean per cell: large dots). Please refer to Fig. S3 for images of all cells that went into this quantification. Additionally, all cells and ROIs can be reviewed on BioImage Archive. One way Anova with Tukey`s multiple comparison test. *** P<0.001, ** P=0.0022. Values are represented as means ± SD (n=10 cells) for each genotype resulting in 1.62 ±0.37, 1.16 ± 0.17, 1.19 ± 0.16, 1.11 ± 0.06 and 1.58 ± 0.39 for G-*B*-G, G^5X^-*B*-G, G-*B*-G^5X^, G^5X^-*B*-G^5X^ and G-*B*-G*, respectively.

Having shown that G-*B*-G associates preferentially with MTs, we then asked which regions in GABARAP, or in G-*B*-G, might be involved. For this purpose, Huh KO cells expressing G^5X^-*B*-G or G-*B*-G^5X^ with a total of five alanine substitutions of N-terminal basic residues (K2A, K13A, R15A, K20A, K23A) either in the first or second GABARAP moiety of the split tandem construct were investigated, as basic residues have been suggested to be part of GABARAP’s tubulin binding motif in former *in vitro* studies. Compared to control cells expressing G-*B-*G, G^5X^-*B*-G or G-*B*-G^5X^ transfected cells expressing constructs with a single intact tubulin-binding motif mostly showed little to no enrichment at MTs as visualized by co-staining with SiR-tubulin (Figure 3A & Fig. S3A-C). Indeed, G^5X^-*B*-G and G-*B*-G^5X^ transfected cells were not distinguishable from G^5X^-*B*-G^5X^ expressing cells, indicating that substituting the basic amino acids in the second GABARAP molecule had no further influence on the result (Figure 3A, C & Fig. S3A-D). Additionally, Huh KO cells expressing G-*B*-G* and co-stained with SiR-tubulin were imaged, permitting investigation of whether lipidation is required for MT association, as the corresponding G-*B*-G* fusion protein is devoid of C-terminal glycine residues suitable for lipidation in both GABARAPs. Interestingly, lipidation deficiency did not appear to inhibit enrichment of G-*B*-G* at MTs, as cells expressing this construct presented themselves indistinguishable from cells expressing the unmodified G-*B*-G (Figure 3B, D & Fig. S3E).

These observations were again substantiated by quantitative analysis. While mean F:C ratios of G-*B*-G and G-*B*-G* expressing cells showed no significant difference (1.62 ± 0.37 and 1.58 ± 0.39), enrichment at filaments compared to adjacent cytoplasm was significantly lower for both G^5X^-*B*-G, G-*B*-G^5X^ (1.16 ± 0.17, 1.19±0.16) and G^5X^-*B*-G^5X^ (1.11±0.06). These findings thus reinforce the conclusion already drawn from the monovalent constructs *B*-G and G-*B* (Fig. S1D, E) and support the idea of avidity-mediated affinity enhancement.

### Tandem GABARAP requires mTagBFP2 for visualization of its microtubule association and cytoskeletal remodeling

**Figure 4:**
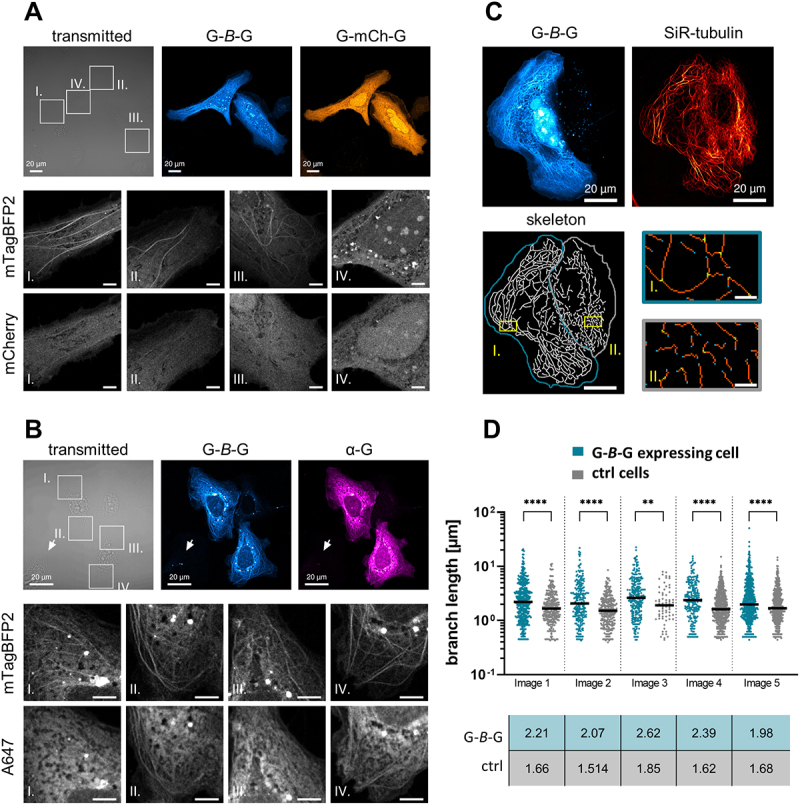
FPs appear sensitive to the MT environment, while G-*B*-G can alter microtubule network organization (**A**) Exemplary image of G-*B*-G and G-*mCh*-G expressing Huh KO cells. The bottom panels show magnifications of four selected ROIs. Scale bars = 5 µm (**B**) Exemplary image of G-*B*-G expressing cells fixed and immunostained with anti-GABARAP polyclonal primary antibody. Arrows indicates exemplary non-transfected control cell. The bottom panels show magnifications of four selected ROIs. Scalebars = 5 µm (**C**) Altered microtubule organization in G-*B*-G expressing Huh KO cells. Top images show exemplary G-*B*-G expressing cell and control cell, both stained with SiR-tubulin (Image 1). Bottom images show skeleton of SiR-tubulin stain in Image 1. The G-*B*-G expressing cell is marked in blue, the control cell in gray. Skeleton was thresholded and dilated for visualization purposes. Exemplary regions are marked in yellow and shown as magnified ROIs (Scale bar = 2 µm) from skeletonized image after tagging of endpoint (blue), junction (green) and slab (orange) pixels for each cell. (**D**) Quantification of branch length from 5 images (see Fig. S4D) each displaying a G-*B*-G expressing and control cell. **** P < 0.0001, ** P=0.0021, Mann-Whitney test. Individual branches are represented including medians, with values in table below the graph.

To our surprise, when changing the FP from mTagBFP2 to mCherry (*mCh*) within the split-tandem construct, no highlighting of filamentous structures occurred in cells expressing G-*mCh*-G (Fig. S4A). As full-length translation and a good expression level of G-*mCh*-G, just as for any other tandem construct, was confirmed (Fig. S4B), we concluded that the behaviour of the GABARAP tandem was influenced by the type of FP selected. Therefore, we also inspected G-*B*-G and G-*mCh*-G co-expressing cells, and selected for those cells that showed a filamentous staining pattern in the blue channel. In such cells, filaments were also faintly visible in the red channel in some regions, though they were hard to distinguish from background (Figure 4A). To better understand this phenomenon, we next made some efforts to visualize MT-associated G-*B*-G independently of mTagBFP2ʹs intrinsic fluorescence, using immunofluorescence. Fluorescence from mTagBFP2, even following fixation, was suitable to select for cells with a pronounced MT-like G-*B*-G pattern. Counterstaining the same cells with a polyclonal anti-GABARAP antibody in combination with an Alexa647-conjugated secondary antibody resulted in a prominent cytoplasmic staining pattern with very limited obvious filament staining particularly in the cell center (Figure 4B, I./III.). However, if one focuses on regions of the flattened cell periphery where less background from freely diffusing G-*B*-G is expected, a filamentous pattern was frequently evident also in the IF-stain (Figure 4B, II./IV.). Since the IF staining pattern obtained was reminiscent of the G-*mCh*-G staining pattern of cells cotransfected with G-*B*-G, we envision the vicinity of MTs as a preferable environment specifically for mTagBFP2 (see discussion for more details).

We finally used the same antibody in an immunofluorescence staining together with an anti-tubulin antibody to investigate what happens when GABARAP is overexpressed without any fusion partner (Fig. S4C). With this setup, no enrichment of GABARAP at MTs occurred, again in accordance with the idea of avidity-based affinity enhancement.

During investigation of G-*B*-G expressing cells, it became apparent that G-*B*-G decorated MTs often appeared strikingly long and curved, with a tendency to even build loops. To analyze this prominent alteration of the MT network, Huh KO cells expressing G-*B*-G were co-stained with SiR-tubulin. Pairs of untransfected cells and cells strongly expressing G-*B*-G were selected and analyzed regarding their MT network organization; we observed clear differences in the network throughout the analyzed cells, with G-*B*-G expressing cells presenting prominently long and curved MTs (Figure 4C). Skeletonization of filamental structures in the SiR channel and subsequent determination of branch length, defined as distance between endpoint and/or junction pixels, showed significantly longer branches for all analyzed G-*B*-G expressing cells (extending up to 50 µm) compared to control cells (Figure 4D, Fig. S4D). Additionally, cytoskeleton bundling parameters, namely coefficient of variance (CV) and skewness, were examined for pairs of G-*B*-G expressing and control cells with similar mean intensities in the SiR-channel (less than 1.6-fold difference, Image 1,2,5). Higher CV and Skewness values for G-*B*-G expressing cells compared to control cells supported the frequent visual observation of high SiR-tubulin intensities associated with pronounced, long MTs (Figure S4E). It is important to note that the altered MT network organization in G-*B*-G expressing cells was not only observable with SiR-tubulin staining, which is known to stabilize MTs, but also in fixed cells stained with an antibody against β-tubulin (Fig. S4F).

### AlphaFold predictions suggest charge-mediated GABARAP enrichment at microtubules rather than a distinct binding mode

**Figure 5:**
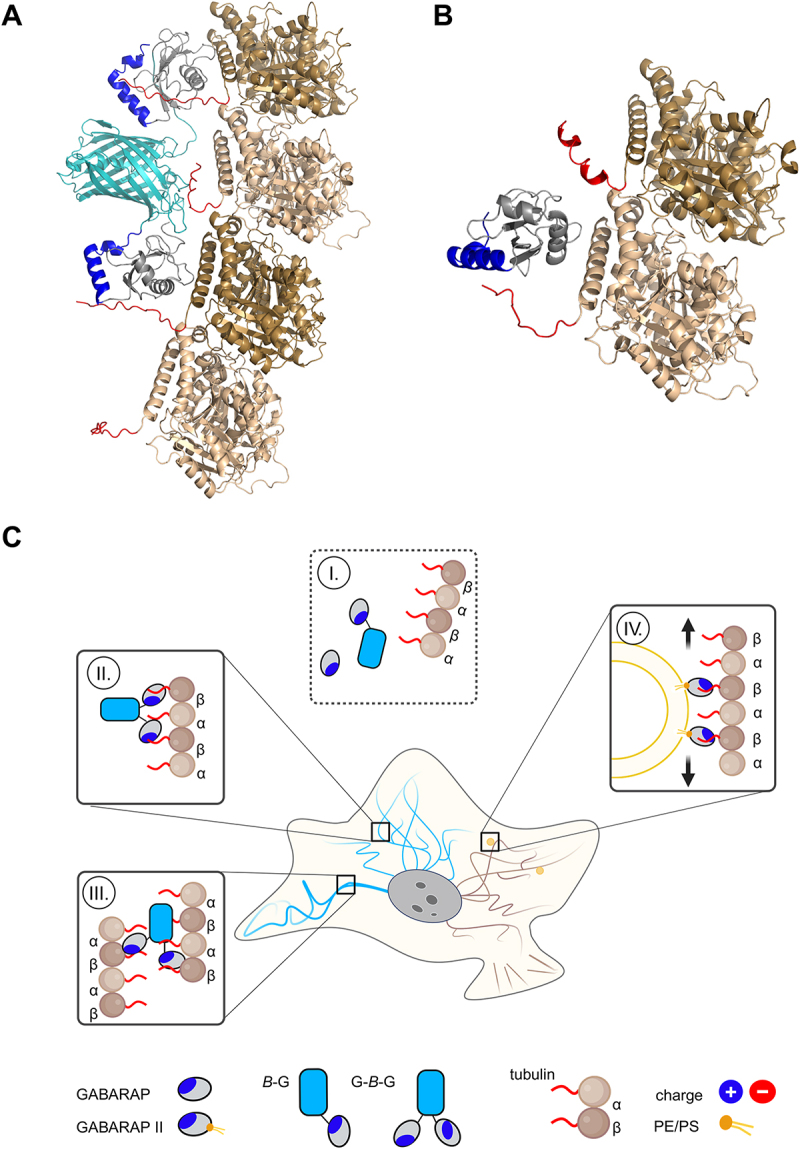
Predictions support a GABARAP-tubulin interaction, although FP and valency of GABARAP likely influence the biological outcome. Complex models of tubulin oligomers with G-*B*-G (**A**) and GABARAP (**B**). This study´s observations along with their possible biological consequences are summarized schematically in (**C**), with more details being explained in the main text. Created with BioRender.com

The presence of two GABARAP molecules in the fusion protein construct G-*B*-G appeared to be the critical factor for MT association. Using ColabFold to predict the structural arrangement of G-*B*-G with a protofilament composed of two tubulin dimers (TBA1A-TBB5), we sought to address the question whether this interaction is sterically plausible. First, orientation of the two GABARAP moieties towards the tubulin chain and of mTagBFP2 away from it was consistently observed, with the GABARAPs typically aligning with the tubulin protofilament in such a way that one protomer is skipped (Figure 5A, Fig. S5A). Despite significant variance among individual models regarding details of the predicted contacts, GABARAP was frequently suggested to associate with the negatively charged C-terminal tail present in both α- and β-tubulin. Due to the inherent disorder of this segment, GABARAP is unlikely to adopt a well-defined orientation relative to the globular tubulin core, as reflected by high PAE scores for intermolecular pairs of residues. With due caution owing to the complexity of the system, these predictions support the idea of electrostatic interactions between G-*B*-G and microtubules. Predictions of untagged GABARAP with a TBA1A-TBB5 dimer also hint at an attraction of GABARAP toward the exposed C-terminal tails of the tubulin monomers (Figure 5B. Fig. S5B). In accordance with the observation in cells, where *B*-G-G showed enrichment at MTs comparable to G-*B*-G, ColabFold predicted a similar charge-dominated tubulin interaction of GABARAP moieties, despite their shorter separation in *B*-G-G (Fig. S5C). However, predictions with untagged GABARAP and tetrameric tubulin did not yield consistent results, possibly indicating a lower interaction propensity. Notably, these calculations do not include the various post-translational modifications described for both GABARAP and tubulin and only represent tubulin dimers and tetramers of specific isotypes, TBB5 and TBA1A. However, both post-translational modifications and isotypes are important for structure and functions of MTs, as well as for interactions with other proteins, possibly including the proposed interaction with GABARAP.

## Discussion

The aim of this work was to exploit novel FP-based tagging strategies to microscopically track GABARAP, a member of the hATG8 family, in living cells, thereby identifying new options for visualizing its functionalities. We propose that in particular GABARAP tandem constructs may expand the scope of current strategies, such as the use of conventional N-terminal FP tags [45,46], or the use of LIR-based sensors [15,47-49]. While particularly N-terminal FP tags provide plenty of options to follow autophagic processes [14], smaller tags (such as HA) or tagless strategies are mandatory in certain situations [26,34]. Taking advantage of avidity through the combination of two GABARAP molecules in one construct, our strategy particularly targets GABARAP activities featuring only moderate interaction strengths for individual binding events.

In detail, we observed differences in the nucleocytoplasmic intensity ratios between G-*B*-G and the conventionally labeled *Y*-G (expressed simultaneously or separately) with an overall higher ratio for *Y*-G. Little is known about the role of GABARAP in the nucleus yet, and pronounced nuclear localization of ATG8s has been interpreted as an artefact in some settings [14]. For LC3B, which is characterized better in this respect, its nuclear pool [50], its differing levels across nuclear compartments [51], and its contribution to nuclear surveillance mechanisms are well described [52-54]; in part these findings may also apply for GABARAP. For instance, GABARAP binds, like LC3B, to lamin B1 [52] and is reported to be sensitive to deacetylase inhibitors [55], which is reminiscent of acetylation-dependent shuttling of LC3B between nucleus and cytoplasm [50]. Notably, in *C. elegans* the GABARAP homolog LGG-1 is involved in modulating lifespan by regulating nuclear dynamics [56]. Higher intensities in the nucleoli compared to the nucleoplasm were observed for G-*B*-G and *B*-G compared to *Y*-G, suggesting that the FP choice and not the number of GABARAPs in the construct is decisive here. *B*-G might be an excellent choice to address GABARAP shuttling between nuclear compartments, technically even in a more direct way than previously reported for LC3B fusions with Venus, a close relative of YFP [53].

The most striking feature of G-*B*-G is its ability to highlight filamentous structures, namely MTs, in the cytoplasm, a behavior hitherto undocumented for conventional FP-tagged GABARAP/ATG8s. Notably, the FP itself significantly influenced the result, as demonstrated for G-*mCh*-G. As we do not have in-depth experimental data on this, we can only speculate on the reasons why mTagBFP2 appears to have a particular effect on the system studied here. In principle, the identity of the FP may influence the preferred relative orientation of the GABARAP moieties in the tandem construct and hence their MT interaction propensity, but this does not seem very likely given the insensitivity to the position of mTagBFP2 (G-*B*-G vs. *B*-G-G). Under certain conditions individual FPs themselves may exhibit an as yet uncharacterized intrinsic tubulin-binding capacity or may interact with certain MT-associated proteins, however, we are not aware of any described MT-binding of free mTagBFP2. In addition, we show that using mTagBFP2 within tubulin-binding incompetent GABARAP tandem constructs does not lead to association with MTs either. Besides a positive contribution of mTagBFP2 to MT association, an absence of negative effects (such as repulsion) compared to other FPs (e.g. mCherry) would also be explain the differences observed. Fluorescent proteins are inherently sensitive to solution conditions, and certain environments are known to affect chromophore functionality and ultimately the measurable fluorescence intensity of some FPs [57-59]. It should be noted, that MTs have highly unusual electrical properties due to the exceptionally negatively charged C-termini of tubulin subunits giving rise to a length-dependent net dipole moment of the entire MT [60]. As one conceptual possibility the vicinity of an MT is suggested to be a confined zone of the cytoplasm with very special characteristics [60,61]. Thus, an alteration of the properties of the FP, locally restricted to the MT zone and maybe differently pronounced depending on the FP used, could also serve as a possible, albeit speculative explanation for our observations. Interestingly, G-*B*-G fluorescence retained a pronounced MT-like pattern even following fixation, while counterstaining with a polyclonal anti-GABARAP antibody resulted in a prominent cytoplasmic stain with only some filaments visible in the flattened cell periphery. Curiously, a recent independent study also revealed different behaviors of mTagBFP (a close relative of mTagBFP2 [62,63]) and mCherry, interestingly in the context of a sensor for tyrosinated MTs [64]. Overall, the question why mTagBFP2 has this peculiar effect on the system studied here is awaiting a definite answer. Nonetheless, our results are significant because they show a striking example of what can in principle apply to any binding study with FP-target protein (e.g. GABARAP) fusion proteins: individual FPs may modulate the degree of visibility of the binding event of interest to different extents, either by differential “interference” with the binding itself (including both positive and negative contributions) or by different modulation of the fluorescence strength (enhancement or attenuation) by the environment of the FP in the bound state.

Nevertheless, there is abundant evidence for a seemingly robust interaction of GABARAP both with tubulin and assembled MTs *in vitro*. GST-GABARAP associates with purified tubulin but not actin, and heterologously expressed GABARAP interacted with *in-vitro* assembled MTs [36], supporting our observation that lipidation is not a prerequisite for G-*B*-G enrichment at MTs in cells. The tubulin-binding region was narrowed down to the first 35 residues of GABARAP [36], and positively charged residues promote GABARAP´s association with MTs through ionic interactions with the negatively charged C-terminal tails of the tubulin monomers [31,36]. This is consistent with our observation of significantly reduced MT association for G^5x^-*B*-G, G-*B*-G^5X^ and G^5X^-*B*-G^5X^, which lack the basic residues K2, K13, R15, K20 and K23 in the first, the second or both GABARAP(s) of the split tandem. While colocalization of GABARAP with MTs has been demonstrated by immunofluorescence under endogenous conditions in Chinese hamster ovary cells more than 20 years ago, utilizing an in-house polyclonal anti-GABARAP antibody [36], this has not been consistently observed by other groups, presumably due to specific properties of the antibodies used. For instance, the epitope of the monoclonal antibody 8H5 developed in our lab [65] overlaps with the proposed tubulin-binding region of GABARAP, preventing detection of the MT-associated fraction. A commercially available polyclonal GABARAP antibody can visualize GABARAP at MTs, though staining is faint and restricted to cells overexpressing G-*B*-G. Notably, NMR experiments probing GABARAP with short peptides derived from the α- or β-tubulin C-terminal tails revealed only moderate affinities (dissociation constants in the 0.1–0.2 mM range) with low specificity [30]. In agreement with these data, in our study constructs with a single GABARAP molecule (*B*-G, G-*B*) did not arrange in filamentous patterns within living cells, while two GABARAPs combined in a tandem reporter (G-*B*-G, *B*-G-G) were required and sufficient to robustly colocalise with MTs in living cells.

It is known that GABARAP can promote MT polymerization *in vitro* and that the first 22 N-terminal residues are sufficient for this purpose [31,35]. In addition, an MT-bundling activity was suspected for GABARAP [66] and for its close relative GABARAPL1 [67]. Shielding of the negatively charged C-terminal tails of tubulin protomers by positively charged patches on MT-associated proteins, as described for the cytoskeleton-associated protein glycine-rich (CAP-Gly) domain of p150^glued^ [68], offers one possible mechanism for these *in-vitro* observations. Likewise, the basic N-termini of GABARAP and GABARAPL1 may neutralize the repulsive negative surface charge of MTs. Interestingly, we repeatedly observed an altered MT network organization including cytoskeleton bundling in G-*B*-G expressing cells, indicating that this GABARAP activity may indeed have significance *in vivo*. Since the G-*B*-G construct offers two tubulin binding sites, it also could bridge individual MTs to form bundles. The bundling promoting properties of the MT associated protein tau have been suggested to be based on a similar mechanism [69]. Remarkably, Nymann-Andersen et al. (2002) already discussed MT binding of GABARAP dimers [70]. How the observed MT network alterations are facilitated in detail, and to what extent the effects observed with the split tandem construct can be translated to wild-type cells expressing GABARAP at endogenous levels remains to be elucidated in future investigations. However, we hypothesize that, if GABARAP-mediated MT changes are relevant at all under endogenous GABARAP levels, they should be confined to well-defined patches of locally high GABARAP density, but are unlikely to produce such global changes in MT organization as observed under G-*B*-G overexpression.

Compared to the relatively controlled conditions *in vitro*, factors influencing MT dynamics and stability in living cells are much more complex. Stability determining factors include the prevalence of tubulin isotypes [71], different post-translational modifications [72-76], and the presence or absence of diverse MT associated proteins (MAPs, [77,78]), which often regulate transport along MTs [79,80]. Kinesin family member 5B (KIF5B), for instance, has been connected to lysosome transport in autophagy, and its depletion led to perinuclear accumulation of autophagosomes in cancer cells [81,82]. In the context of insulin vesicle transport a connection between KIF5B and GABARAP has been proposed, as GABARAP appears to promote vesicle trafficking by KIF5B [83]. Considering our results, it is conceivable that GABARAP not only presents transport vesicles to KIF5B by connecting vesicles and MTs and thereby stabilizing the kinesin-cargo complex, but at high local concentrations additionally stabilizes MTs and thereby supports KIF5B binding and corresponding anterograde vesicular transport.

Given that members of the LC3 subfamily of ATG8 proteins have been first described as light chains of the MT interacting proteins MAP1A and MAP1B [84,85], further connections between ATG8s and MTs do not seem far-fetched. Accordingly, the connection between autophagy and the cytoskeleton has been extensively studied and reviewed [86-89]. Regarding autophagy-unrelated functions, an interplay between GABARAP and MTs has been suggested for the anterograde transport of e.g. the GABA_A_, angiotensin II type 1 and κ opioid receptors [46,90,91]. The LC3B-mediated transport of melanosomes along MTs and their detachment from MTs by ATG4B-mediated delipidation is another example of this connection [92].

In summary, while untagged and conventionally tagged GABARAP, e.g. *B*-G, do not visibly associate with MTs even under overexpression (Figure 5C-I), G*-B*-G accumulates on MTs (Figure 5C-II) owing to the bivalent nature of the construct and the yet to be defined role of mTagBFP2 in this process. Drechsler et al (2019) recently reported that multivalence is a critical property of MAPs, conferring MT-bundling abilities, optionally by bridging individual MTs which can lead to bundling [93]. However, as bundling could be an artificial activity of GABARAP tandems (Figure 5C-III), this aspect should be viewed with caution. GABARAP-decorated vesicles such as autophagosomes and endolysosomal vesicles [94], however, could present multiple GABARAPs in proximity to MTs, resulting in significant avidity through multivalence. Such multiple GABARAP-MT interactions might therefore assist in keeping those vesicles on the MT track, possibly promoting their transport (Figure 5C-IV).

As both impaired MT dynamics and defective autophagy as well as their interplay have been linked to human disease [95-97], understanding how GABARAP and other ATG8 proteins influence MT stability and related processes may also provide novel insights into pathophysiology and suggest improved strategies of intervention. While our results employing a GABARAP split tandem construct within living cells are not necessarily transferrable to the functionalities of endogenous GABARAP, our results may serve as a motivation to re-visit the GABARAP-microtubule interaction described more than 20 years ago using today’s techniques.

## Abbreviation

ATG8: autophagy related protein 8

FP: fluorescent protein

GABARAP: γ-aminobutyric acid type A receptor-associated protein

Icorr: Index of correlation

KO: knockout

LDS: LIR docking site

LIR: LC3-interacting region

MAP1LC3/LC3: microtubule associated protein 1 light chain 3

MAP: microtubule associated protein

MT: microtubule

nMDP: normalized mean deviation product

ROI: region of interest

SiR: Silicon rhodamine

## Supplementary Material

Ueffing_SI_one_PDF.pdf

## Data and material availability

Original imaging data is available on BioImage Archive at https://www.ebi.ac.uk/biostudies/bioimages/studies/S-BIAD952. All described plasmids are deposited at Addgene.
