# Supplementary material for: Highlighting the hidden: monitoring the avidity-driven association of a fluorescent GABARAP tandem with microtubules in living cells
Source: Autophagy Rep. 2024 May 16;3(1):2348899. doi: 10.1080/27694127.2024.2348899 (PMC11864619; doi:10.1080/27694127.2024.2348899)
Supplement: Ueffing_SI_one_PDF.pdf [file KAUO_A_2348899_SM8472.pdf]

SI Methods

Immunocytochemistry

Transfected Huh-7.5 *GABARAP* SKO cells shown in Fig.S4C were washed in Cytoskeleton buffer (CB, 150 mM NaCl, 5 mM $MgCl_2$, 5 mM EGTA, 5 mM glucose, 10 mM MES, 1 g/L streptomycin) and fixed in 3.7% PFA in CB for 20 min at 37°C. The reaction was stopped with 30 mM glycine in CB for 5 min, like all following steps, if not indicated otherwise, at room temperature. The cells were permeabilized with 2% Triton-X-100 for 2 min, washed thrice for 5 min with CB and non-specific binding sites were blocked with 5% (w/v) milk powder in CB for 30-45 min. Afterwards, cells were incubated with primary antibodies against GABARAP (anti-GABARAP rabbit polyclonal [Proteintech, 18723-I-AP) diluted 1:100 in CB containing 1% (w/v) milk powder and against α-tubulin (anti-α-tubulin, rat monoclonal clone YL1/2, [Signa-Aldrich, MAB1864]) diluted 1:200 in CB containing 1% (w/v) milk powder for 1 h at 37°C. Next, cells were washed thrice in CB and stained with secondary antibodies (anti-rabbit-Alexa647 [abcam, ab150083] and anti-rat-CY3 [Jackson Immunoresearch, 112-165-006]) diluted 1:200 in CB containing 1% (w/v) milk powder for 45 min at 37°C. After 2 washes in CB, and one in PBS (137 mM NaCl, 2.7 mM KCl, 1.8 mM KH_2PO_4, 10 mM Na_2HPO_4, pH 7.4), cells were imaged and stored in PBS containing 0.05% (w/v) sodium azide. Transfected Huh-7.5 *GABARAP* SKO cells shown in Fig.S4F were fixed for 10 min with precooled methanol and subsequently for 1 minute with precooled acetone, both at -20°C. After two washes with PBS, non-specific binding sites were blocked with 5% (w/v) BSA in PBS for 1 h. Cells were incubated with anti-β-tubulin-CY3 mouse monoclonal [Sigma Aldrich, C4585]) diluted 1:100 in PBS containing 1% (w/v) BSA (AppliChem, A1391) and 0.3% (v/v) Triton X-100 (AppliChem, A4975) for 2 h or overnight and afterwards washed twice with PBS. Finally, cells were imaged and stored in PBS containing 0.05% (w/v) sodium azide.

Immunoblotting

Transfected Huh-7.5 *GABARAP* SKO cells were harvested by trypsination, washed once in PBS and lysed with NP40 buffer (20 mM Tris HCl, 200 mM NaCl, 1 mM EDTA, 0.5% NP40, 1 mM PMSF and 1x Halt Protease and Phosphatase inhibitor [Thermo Fisher Scientific, 78442]) by incubation on ice for 30 min and vigorous pipetting every 10 min. Insoluble cell parts were sedimented by centrifugation for 10 min at 17,000 x g. Total protein concentration of supernatant was determined by BCA assay (Thermo Scientific, 23225). Samples were separated by SDS-PAGE using precast stain-free gels (Bio-Rad Laboratories, 4568124), transferred to 0.2 µM PVDF membranes (Bio-Rad Laboratories, 1704156) and blocked in 5% BSA (AppliChem, A1391) in TBS-T (136 mM NaCl, 2.7 mM KCl, 24.7 mM Tris-HCl, pH 7.4, 0.05% Tween-20 [Applichem, A4974]) for 1 h. Membrane was incubated with primary anti GABARAP antibody (Cell Signaling Technology, 13733) overnight at 4°C, washed 3 times with TBS-T, incubated with secondary antibody (goat anti-rabbit, HRP-conjugated [Dako, P0448]) for 1 h at room temperature and again washed thrice with TBS-T. Signals were visualized with Clarity western ECL substrate (Bio-Rad Laboratories, 1705061).

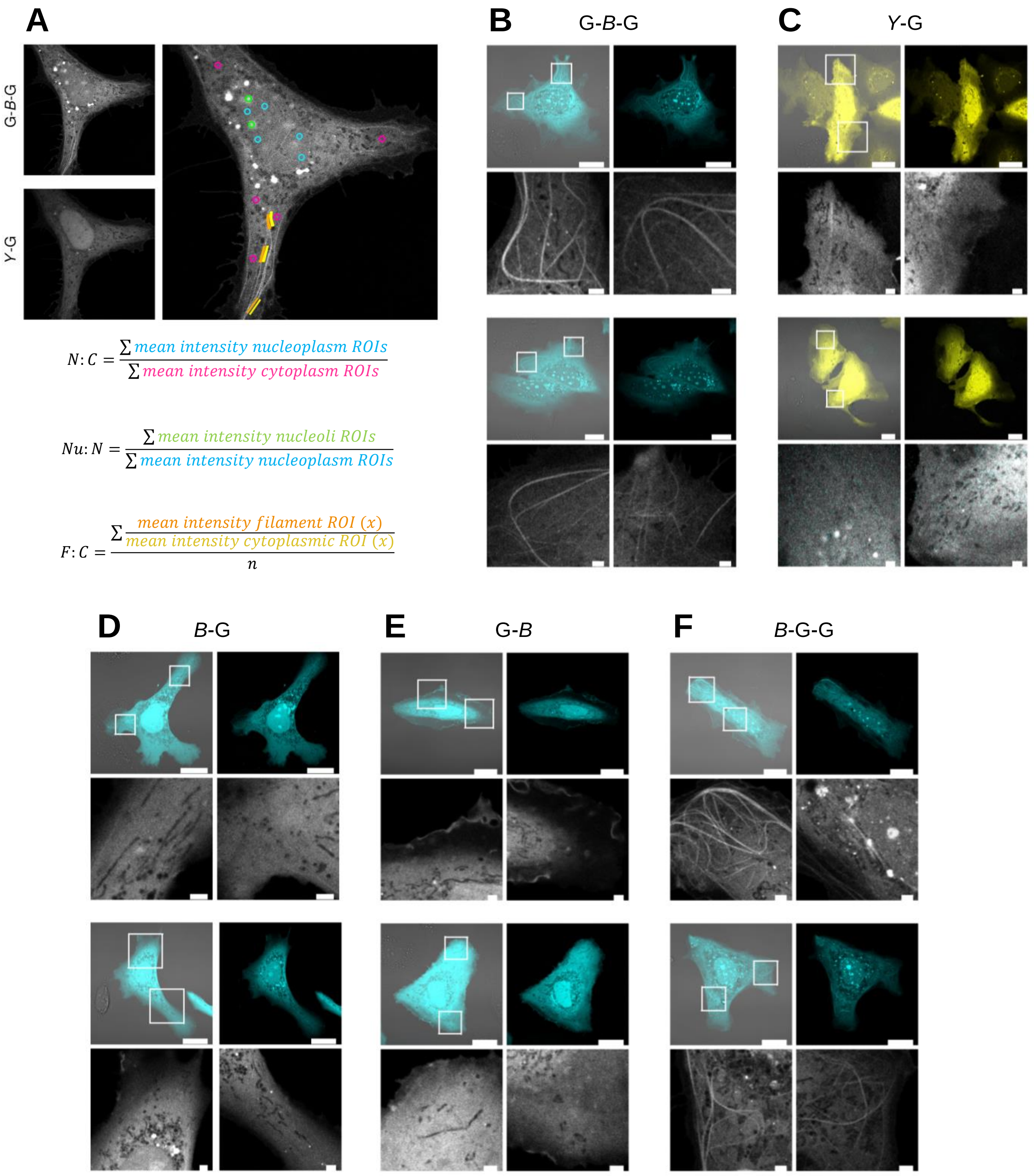

Figure S1. (**A**) Example of cytoplasmic, nucleoplasmic, nucleoli and filament ROIs in a Huh KO cell expressing G-*B*-G and *Y*-G as well as the formulas used for calculation of N:C, Nu:N and F:C ratios. All images and ROIs of cells analyzed accordingly can be found on BioImageArchive. (**B-F**) Live cell images of Huh KO cells expressing G-*B*-G (**B**), *Y*-G (**C**), *B*-G (**D**), G-*B* (**E**) *B*-G-G (**F**). Whole cells (merge with transmitted and G-*B*-G channel) as well as detailed ROIs are shown for two cells per construct. Scale bars represent 20 µm for whole cells and 2 µm for zoom ins.

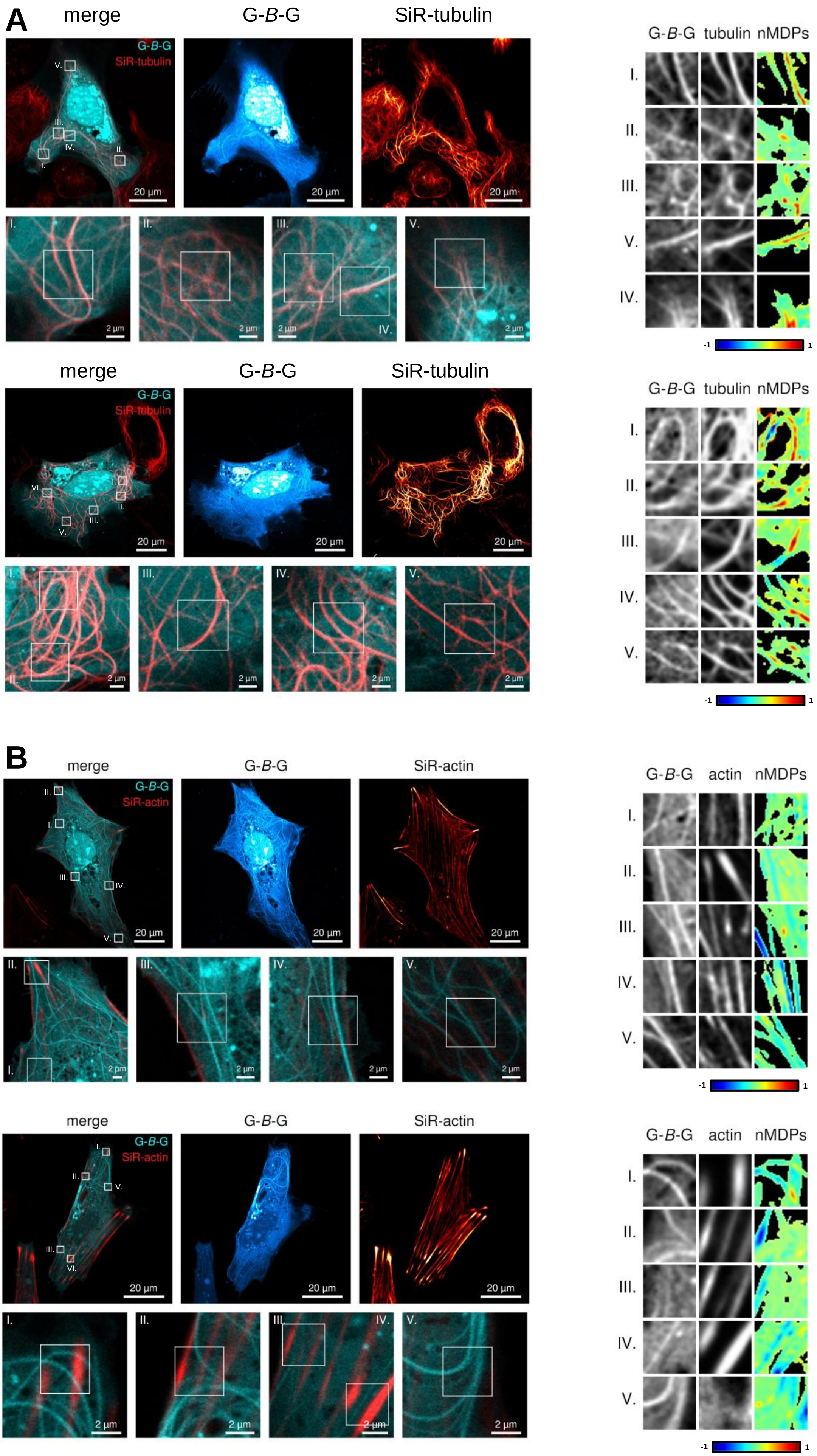

Figure S2. Continued on the following page.

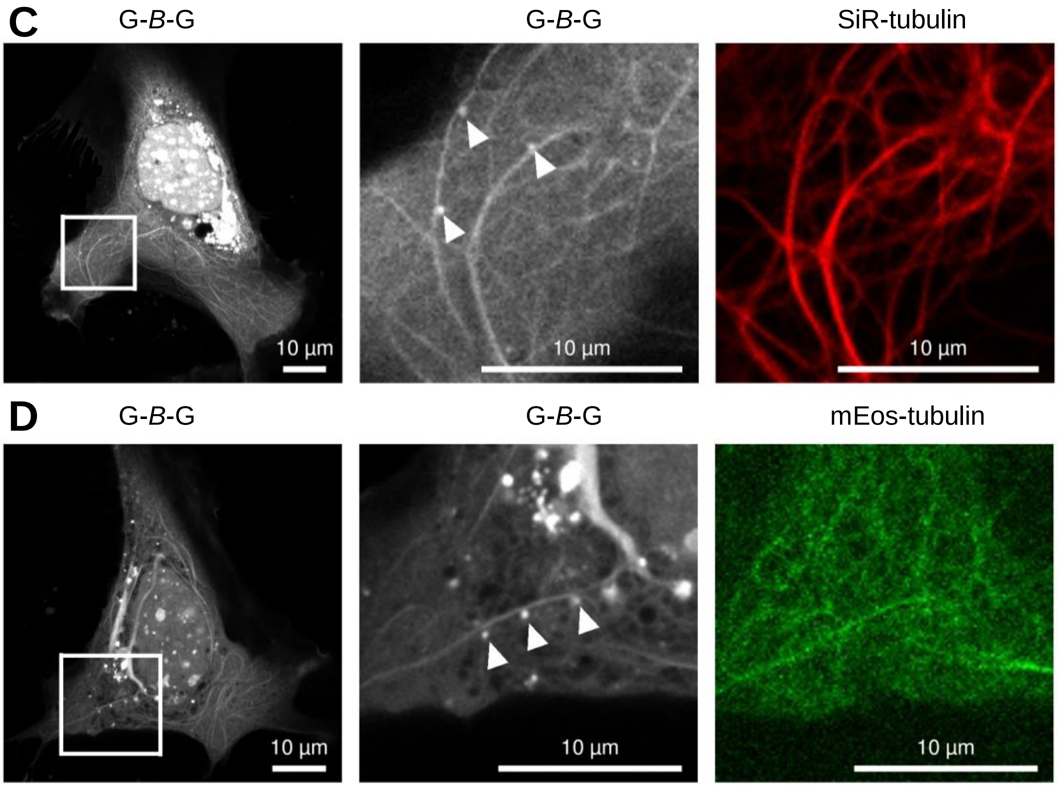

Figure S2. Live cell images of Huh KO cells expressing G-*B*-G and stained either with SiR-tubulin (**A**) or SiR-actin (**B**) together with their colocalization colormaps. The corresponding Icorr values are included within the graph given in Fig. 2D. (**C-D**) Live cell images of Huh KO cells expressing G-*B*-G and either co-stained with SiR-tubulin (**C**) or co-transfected with a plasmid encoding mEos-tubulin (**D**) Zoom in images show microtubules decorated with G-*B*-G positive puncta, likely transport vesicles.

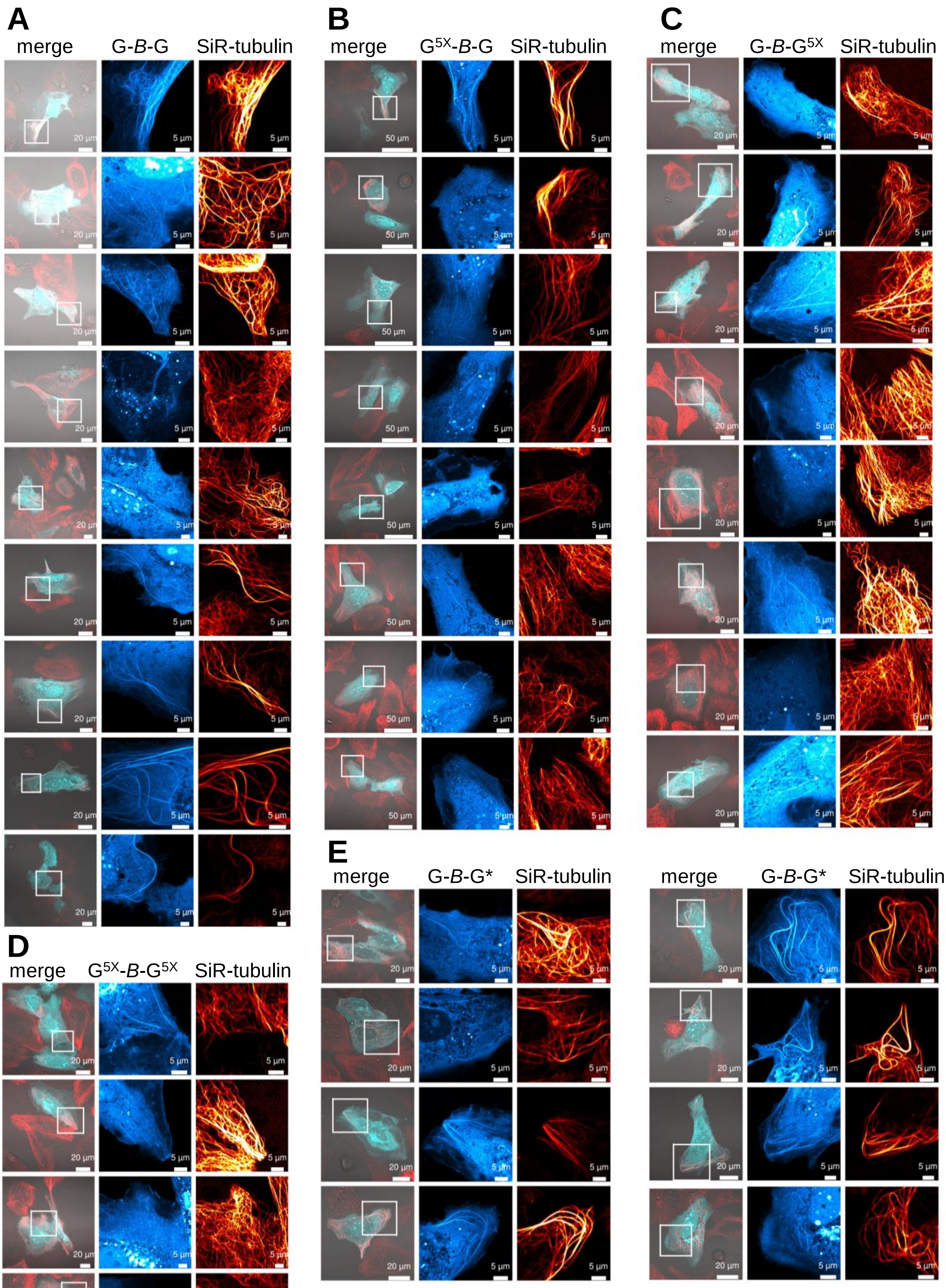

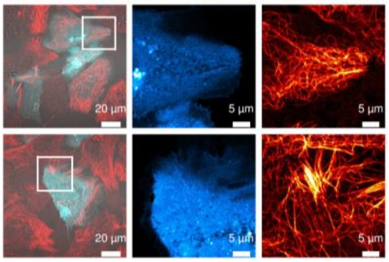

Figure S3. Live cell images of Huh KO cells expressing G-*B*-G (**A**), G^{5X}-*B*-G (**B**) G-*B*-G^{5X} (**C**) G^{5X}-*B*-G^{5X} (**D**) or G-*B*-G* (**E**) and co-stained with SiR-tubulin. All presented cells were used for determination of F:C ratios shown in Figure 3D. Corresponding ROIs for quantification can be accessed on BioImageArchive.

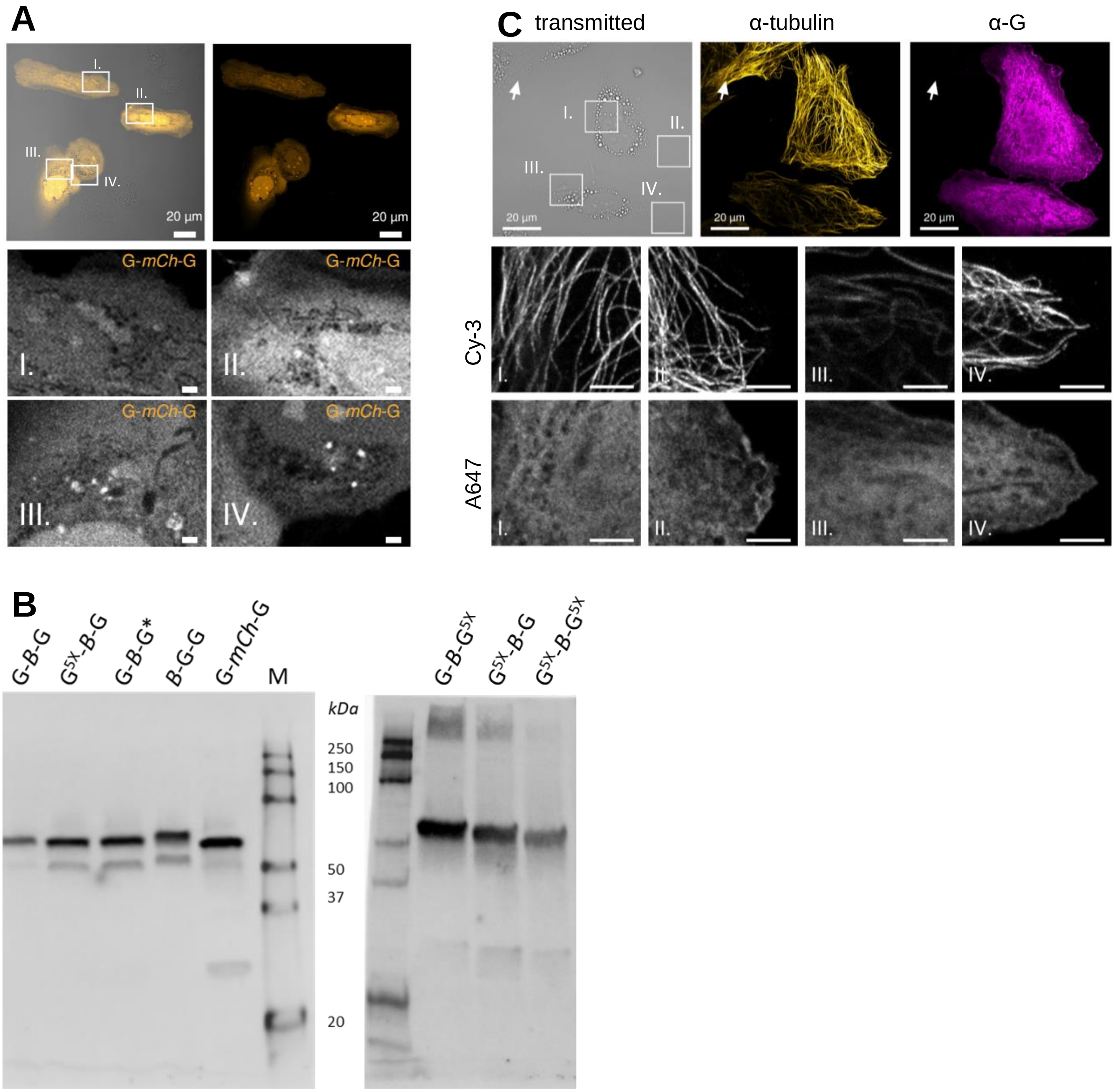

Figure S4. Continued on the following page.

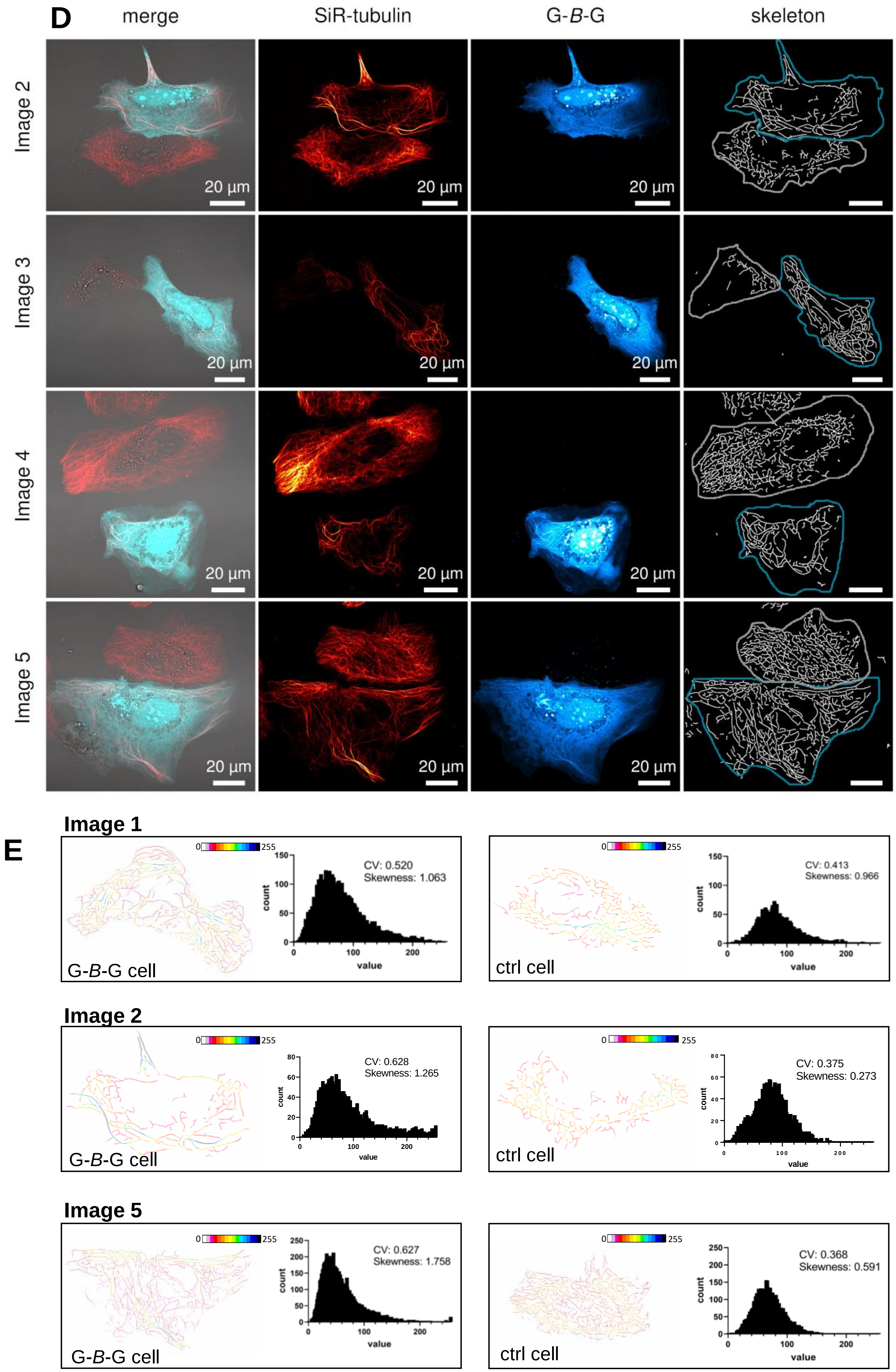

Figure S4. Continued on the following page.

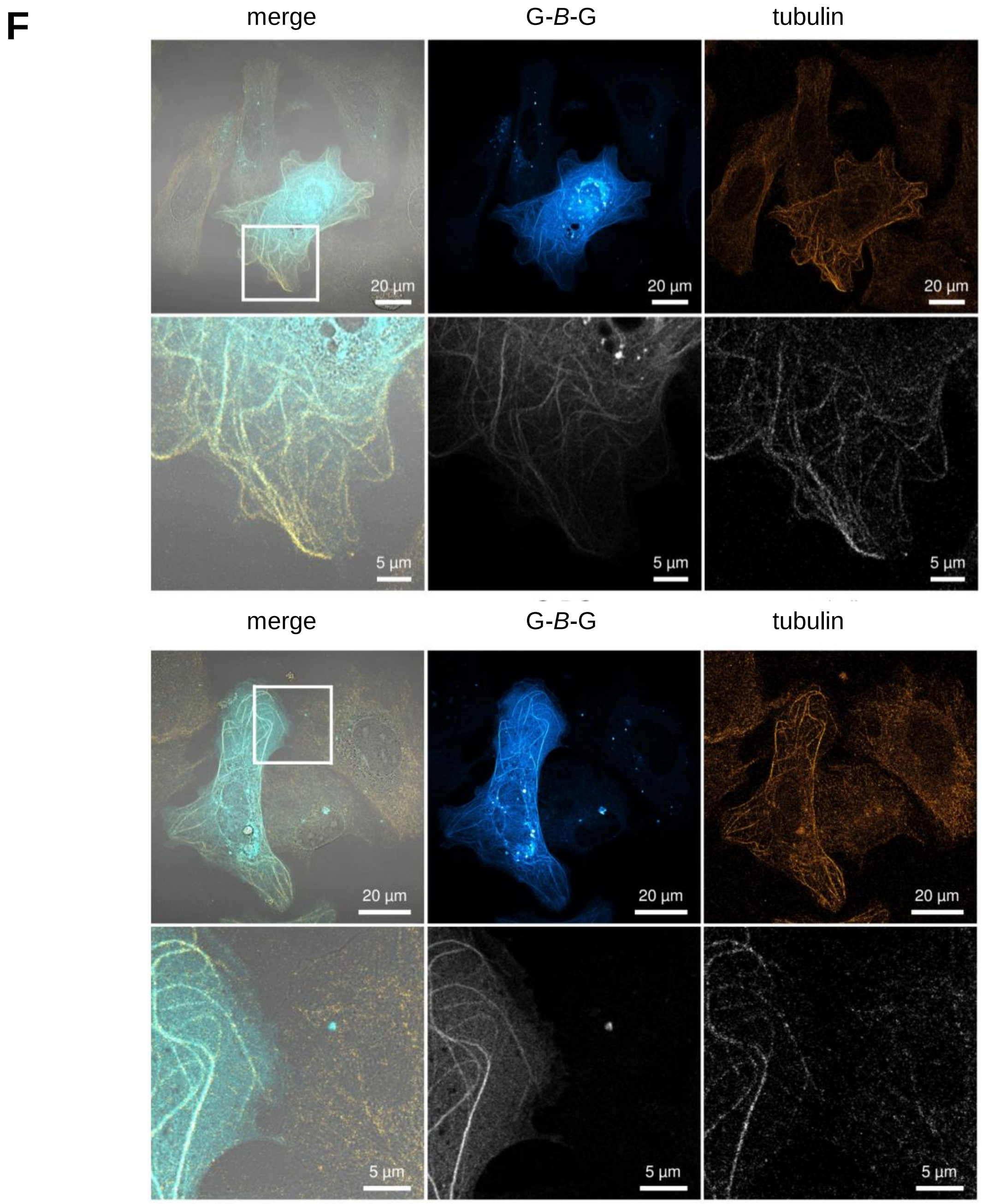

Figure S4. (**A**) Huh KO cells expressing split tandem construct G-*mCh*-G. The bottom panels show magnifications (Scale bars = 2 µm) of four selected ROIs. (**B**) Full length expression of different split tandem constructs (G-*B*-G, G^{5X}-*B*-G, G-*B*-G*, *B*-G-G, G-*mCh*-G and G-*B*-G^{5X}, G^{5X}-*B*-G (shown on both membranes), G^{5X}-*B*-G^{5X}) was confirmed by Immunoblot from whole cell lysates of Huh KO cells transfected with the respective plasmids and staining with GABARAP antibody (Cell Signaling Technology, 13733). The most intense bands were detected at the expected size of the construct at approximately 55 kDa. The signal at about 25 kDa is unlikely to contribute to fluorescence signal in cells. Ladder: BioRad, #1610375. (**C**) Huh KO cells expressing GABARAP (without tag), fixed and stained with primary antibodies against tubulin and GABARAP (Sigma-Aldrich, MAB1864 & Proteintech, 18723-I-AP). Arrows indicate exemplary non-transfected control cell. The bottom panels show magnifications (Scale bars = 5 µm) of four selected ROIs for both channels. (**D**) Images of Huh KO cell pairs, one expressing G-*B*-G and a second untransfected control cell, stained with SiR-tubulin. Skeletons, thresholded and dilated for visualization, for analysis of branch length as shown in Figure 4C are displayed. (**E**) For image 1, 2 and 5, color coded skeletons according to tubulin intensity values and corresponding cytoskeleton bundling parameters are displayed (**F**) Images of fixed Huh cells transiently transfected with plasmid encoding G-*B*-G and stained with an antibody against tubulin. Cells showing strong G-*B*-G expression present an altered tubulin staining pattern.

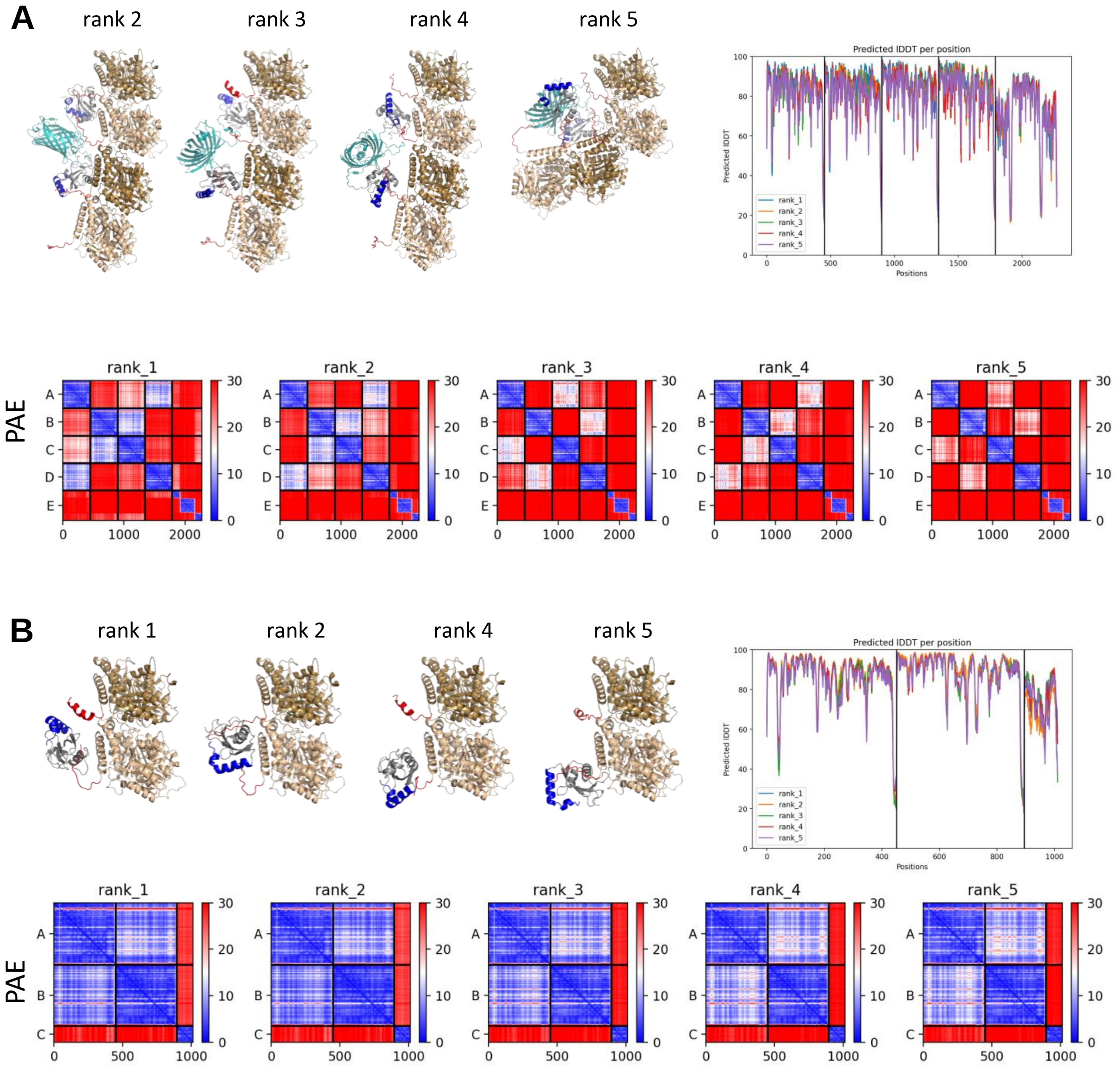

Figure S5. Continued on the following page.

C

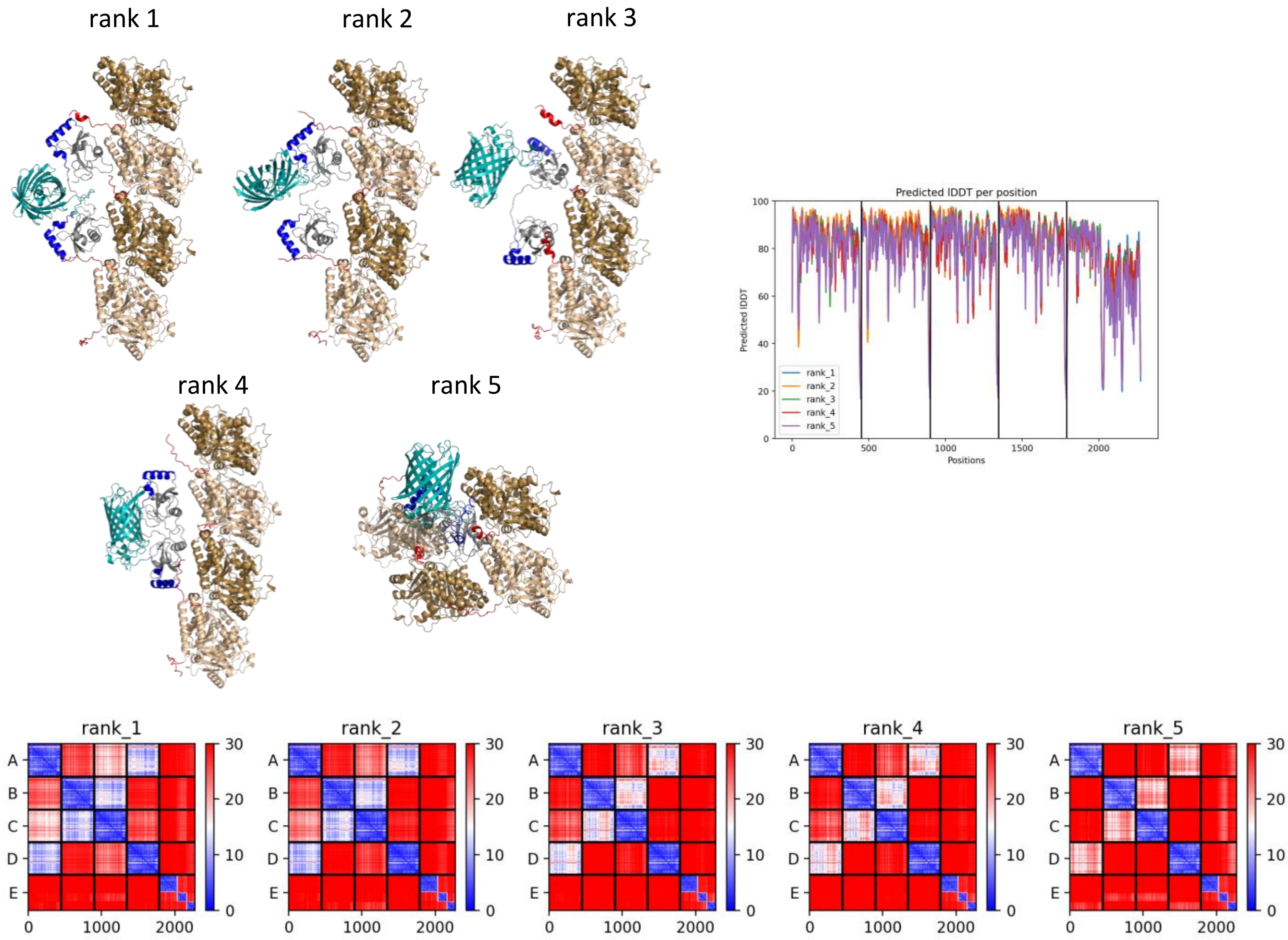

Figure S5. Additional Colabfold models and their corresponding ranks as well as well as per residue confidence values, namely predicted local difference distance test (pIDDT) and predicted aligned error (PAE) for models presented in Figure 5, namely G-*B*-G with a TBB5 and TBA1A tetramer (**A**) and GABARAP with TBB5 and TBA1A (**B**). Additionally, models for *B*-G-G with a TBB5 and TBA1A tetramer is shown (**C**).
